# Cerebrolysin as an adjuvant therapy after mechanical thrombectomy in large vessel occlusion cardioembolic stroke: a propensity score matching analysis

**DOI:** 10.3389/fneur.2025.1510284

**Published:** 2025-02-13

**Authors:** Ahmed ElBassiouny, Mohamed S. A. Shehata, Amr S. Zaki, Rady Y. Bedros, Ayman Hassan El-Sudany, Azza Abdel Nasser

**Affiliations:** ^1^Department of Neurology, Faculty of Medicine, Ain Shams University, Cairo, Egypt; ^2^Faculty of Medicine, Zagazig University, Zagazig, Egypt; ^3^Egyptian Fellowship of Neurology, Ministry of Health, Cairo, Egypt

**Keywords:** Cerebrolysin, mechanical thrombectomy, cardioembolic ischemic stroke, cerebroprotection, endovascular recanalization

## Abstract

**Introduction:**

Endovascular recanalization therapy has demonstrated considerable efficacy in the treatment of acute ischemic stroke (AIS). However, not all patients appear to benefit on the long term from this therapy. No studies have assessed the role of Cerebrolysin following mechanical thrombectomy (MT). The present study was conducted to evaluate the safety and efficacy of Cerebrolysin as add-on treatment to MT in patients with cardioembolic AIS.

**Methods:**

This study evaluated 150 patients admitted to the stroke unit. Data were prospectively collected from 75 patients with cardioembolic AIS and National Institutes of Health Stroke Scale (NIHSS) ≥10, who underwent successful MT ± recombinant tissue plasminogen activator (rt-PA). Patients fulfilling inclusion criteria were consecutively enrolled and treated with Cerebrolysin at a daily dose of 30 ml for 14 days, with treatment initiated within 8 h following MT. Patients were compared with a historical control group of 75 well-matched patients who underwent MT ± rt-PA but did not receive Cerebrolysin. The primary outcome measure was a favorable modified Rankin Scale (mRS = 0–2) at day 90. Secondary parameters included the NIHSS, the Montreal Cognitive Assessment (MoCA), the rate of hemorrhagic transformation, mortality, and adverse events. Propensity score matching was performed to match the variables between the compared groups.

**Results and discussion:**

The overall results demonstrated that patients treated with Cerebrolysin exhibited a significantly higher proportion of mRS scores of 0–2 at day 90 (64% vs. 34.7%) in comparison to the control group. This finding was consistent with lower NIHSS and mRS scores at all study visits, and a lower any hemorrhagic transformation rate (20% vs. 57.3%). Furthermore, the logistic regression analysis revealed that patients with favorable mRS scores were less likely to undergo hemorrhagic transformation (odds ratio = 2.75, 95% confidence interval = 1.17, 6.45; *p* = 0.002). The administration of Cerebrolysin as an add-on treatment resulted in a significant benefit for AIS patients following MT, characterized by an improvement in mRS and NIHSS scores, along with a reduced rate of hemorrhagic transformation. The administration of Cerebrolysin was safe and well tolerated. Further studies are required to confirm these results.

## 1 Introduction

Acute ischemic stroke (AIS) is a significant global health concern, given its high morbidity and mortality rates worldwide (1). In 2015, a major advancement in the treatment of AIS was observed following the publication of groundbreaking randomized controlled trials (RCTs) that compared medical and endovascular treatments. These studies demonstrated the efficacy of mechanical thrombectomy (MT) for large vessel occlusion (LVO) in reducing mortality and improving outcomes (2–4). Of particular note is the finding that the number needed to treat fell within the range of 3–7 individuals (5). The positive outcomes following MT can be attributed to technical advancements and refined patient selection criteria, including perfusion and collateral status assessment (6).

Despite the advancement in MT, a marked discrepancy persists between effective recanalization and the restoration of pre-stroke functional abilities. The mortality rate of 15.3% remains a cause for concern. Furthermore, within a 3-month period, only 46% of individuals with anterior circulation AIS who underwent endovascular therapy attained functional independence (7). In addition, studies have demonstrated significant variability in the long-term follow-up outcomes of thrombectomy, with certain aspects of this treatment, such as the optimal time window, yet to be fully optimized (8).

In view of the aforementioned uncertainties, it is imperative to concentrate not only on advancements in vascular recanalization, but also on cerebroprotection, which refers to strategies to protect viable brain tissue in the acute phase of stroke to minimize infarct growth and preserve neurological function. Consequently, exploring rapid and effective cerebroprotection strategies to complement MT will be crucial in enhancing the prognosis of post-stroke patients (9, 10).

Research in the field of cerebroprotection and neurorecovery in stroke has yielded encouraging results in both preclinical and clinical settings. Despite the promising experiences with various agents, their translation into human application has been challenging due to several factors, such as the absence of continuous reopening of the blocked vessel, delayed intervention, insufficient dosage, and inconsistencies in research methodologies. Consequently, there is necessity for a paradigm shift in modern stroke treatment from pure neuroprotection to neurovascular protection, with the recognition of the critical roles of neurovascular unit elements (11, 12).

Cerebrolysin, a neurotrophic peptidergic preparation, has been demonstrated to exert multifactorial cerebroprotective effects. It enhances cellular survival, and it has been shown to inhibit glutamate excitotoxicity and to counter the formation of free radicals and pro-inflammatory mediators such as TNF-α, IL-1β, IL-6, and NF-κB (11). While the literature consistently demonstrates the pleiotropic and multimodal activity of Cerebrolysin in both *in vitro* and animal studies, there is a paucity of research exploring its efficacy as an adjunct therapy to reperfusion therapy in AIS (12). In an experimental rat model with temporary occlusion of the middle cerebral artery, the administration of Cerebrolysin 3 h following ischemia substantially decreased the volume of tissue damage. This reduction was attributed to the inhibition of neuroinflammation *via* the activation of the CREB/PGC-1α pathway and the suppression of free radical formation, promoting long-term functional recovery (13). A previous pilot trial involving 44 severe stroke patients (NIHSS > 8) treated with futile reperfusion therapy found that those receiving Cerebrolysin (30 mL/day for 14–21 days) showed a trend toward better outcomes (mRS 0–3) at 12 months compared to controls (70% vs. 48%; *p* = 0.1) (14). Another recent clinical trial reported that Cerebrolysin significantly reduced the rate of symptomatic hemorrhagic transformation (OR 0.248, 95% CI 0.072–0.851; *p* = 0.019) (15).

The hypothesis of this study was that the administration of Cerebrolysin within 8 h following the onset of stroke, in subjects who met specific clinical and radiological criteria, had the potential to enhance the efficacy of MT. The initiation of cerebroprotective effects and the prevention of reperfusion injury by this multimodal approach is expected to facilitate post-stroke recovery. The selection of Cerebrolysin was based on its recognized pharmacological attributes, its capacity to penetrate the blood-brain barrier (BBB), its established safety profile, and the encouraging outcomes observed in preclinical studies and clinical trials. The present study assessed the efficacy and safety of Cerebrolysin as a supplementary therapy to MT in both the acute and subacute recovery stages among individuals with cardioembolic AIS.

## 2 Materials and methods

### 2.1 Ethics statement

The present study was granted approval by the Ethical Committee at Ain Shams University in Cairo, Egypt (approval number: FAMASU R85/2024). The study was conducted in strict accordance with the Strengthening the Reporting of Observational Studies in Epidemiology (STROBE) guidelines (16) (Supplementary material) and followed the principles of the Declaration of Helsinki. Written informed consent was obtained from patients in the Cerebrolysin group prior to MT. For participants lacking decision-making capacity, consent was obtained from a legal representative. For the historical control arm, a waiver of consent was granted since the data were anonymized and de-identified prior to analysis, posing no risk to patients, ensuring no impact on participants' rights, involving no additional interventions or contact, and avoiding potential distress to the patients or their family members (17).

### 2.2 Study design and setting

This study was a prospective cohort study with a historical control group. Patients with cardioembolic AIS, which accounts for at least 20% of all ischemic strokes (18), and large vessel occlusion (LVO) who underwent MT were admitted to the stroke unit at Ain Shams University Hospital between October 2021 and January 2024.

#### 2.2.1 Patient population

Patients diagnosed with cardioembolic stroke met the following criteria: at least one cardiac source identified for the embolus and no significant vascular abnormality such as severe atherosclerosis or stenosis in the large ipsilateral arteries associated with the cerebral infarction. Of the 150 patients included in the analysis, 75 patients in the study group received Cerebrolysin within 8 h of the MT, while 75 patients in the control group underwent MT only (identified from medical records between June 2017 and February 2021). Cerebrolysin was administered within 8 h of MT. It is crucial to initiate cerebroprotective measures in the early phase after an AIS while the salvageable brain tissue (penumbra) is still viable (19). Both groups were matched by the occlusion location, age (within ±5 years), co-morbidities, baseline mRS (0–2 or 3–5), initial NIHSS ≥10, initial ASPECT ≥6 (before MT), onset to reperfusion time (within ±2 h), and employing bridging therapy with rt-PA.

The inclusion criteria involved: (1) patients with cardioembolic AIS demonstrating evidence of LVO, (2) patients who received Cerebrolysin infusion within 8 h after successful MT (defined as substantial reperfusion of the affected territory, with a modified Thrombolysis in Cerebral Infarction (mTICI) score of 2b or 3 at the end of the procedure) with or without IV rt-PA administration, (3) age between 18 and 80 years, and (4) a baseline NIHSS score of ≥10, as this is indicative of moderate-to-severe stroke severity, often associated with LVO and higher hemorrhagic transformation. Exclusion criteria encompassed: (1) patients older than 80 years, (2) individuals with epilepsy, (3) pregnant or lactating women, (4) patients with terminal illnesses, or a life expectancy of ≤ 6 months, and (5) individuals with premorbid cognitive impairment, and (6) patients with incomplete medical records.

### 2.3 Treatment

Patients in the study group received an intravenous infusion of 30 ml of Cerebrolysin diluted with 100 ml of 0.9% normal saline. This was administered promptly after thrombectomy, within 8 h of completion of MT. It was then given once daily for a duration of 14 days. This specified dose of Cerebrolysin was administered routinely according to the stroke unit guidelines (15, 20). All patients received standard stroke treatments, which encompassed antiplatelet or anticoagulation medications and control for hypertension, hyperlipidemia, and hyperglycemia. The treatment of patients qualifying for MT followed international guidelines (21, 22). MT was performed using stent retrievers, aspiration, or a combination of these techniques (23), *via* groin access by experienced operators familiar with the MT procedures. The choice of anesthesia, whether local or general, was at the operator's discretion. The MT procedures were consistent across the Cerebrolysin and historical control groups. The mTICI score was used to evaluate reperfusion following thrombectomy. The scale ranges from 0 (no reperfusion) to 3 (complete reperfusion of the previously occluded target artery, with no visible occlusion in distal branches). Intermediate scores include: 1 (limited distal branch filling with slow or minimal reperfusion beyond the initial occlusion) and 2a (reperfusion of less than half of the affected artery territory, such as two major divisions of the MCA and their branches). Bridging therapy with rt-PA followed current guidelines (0.9 mg/kg) (24).

### 2.4 Outcome parameters

The primary outcome parameter was the mRS (score of 0–2) at day 90. Secondary outcome parameters included the mRS and NIHSS at days 14, 30, and 90, the Montreal Cognitive Assessment (MoCA) at day 90, and the symptomatic and asymptomatic hemorrhagic transformation and mortality. The MoCA was used to establish a baseline cognitive profile of the patients, ensuring that any cognitive changes observed later could be attributed to the treatment and recovery process rather than pre-existing conditions or procedural effects. Safety parameters assessed lab values and adverse events.

### 2.5 Neuroimaging

Cardioembolic stroke, defined according to the TOAST criteria (25), has a higher bleeding risk (26). Therefore, this study also aimed to assess the impact of Cerebrolysin on specific subtypes of hemorrhagic transformation: hemorrhagic infarction (HI) and parenchymal hematoma (PH). On a computed tomography (CT) scan, HI appears as a heterogeneous hyperdensity within the ischemic infarct zone, whereas PH is a more homogeneous and dense hematoma with a mass effect. Both HI and PH have two subtypes: HI type 1 (HI1) presents as small, scattered hyperdense petechiae, and HI type 2 (HI2) shows confluent hyperdensity throughout the infarct zone without mass effect. PH type 1 (PH1) is defined as a homogeneous hyperdensity occupying < 30% of the infarct zone with some mass effect, while PH type 2 (PH2) occupies more than 30% of the infarct zone and causes significant mass effect (27, 28). Symptomatic hemorrhagic transformation was defined as the presence of hemorrhage on a CT scan, along with neurological deterioration, indicated by an increase of more than 4 points in the NIHSS (29). Asymptomatic hemorrhagic transformation was defined as radiological evidence of hemorrhage detected on follow-up imaging that was not associated with any new or worsening neurological symptoms. The neuroradiological acute stroke protocol mandates brain non-contrast CT (NCCT) for hemorrhage exclusion, magnetic resonance imaging, magnetic resonance angiography, followed by CT angiography to identify LVO status in eligible patients. We evaluated NCCT images at 24 h (all patients), 1 week, and 2 weeks after MT. Early ischemic indicators were assessed using the Alberta Stroke Program Early CT Score (ASPECTS) on baseline (30). Intracranial hemorrhages were diagnosed *via* control NCCT at 24 h or later for neurological deterioration (a ≥4-point NIHSS score increase from baseline and PH2).

### 2.6 Sample size

The sample size was calculated using G^*^Power 3.1.9.7 software. The favorable primary outcome rate was assumed to be 85% for the Cerebrolysin group and 60% for the control group, based on the findings of previous MT trials (3, 4, 31). With an alpha level of 5%, a total of 150 patients, 75 per group, was required to achieve 80% power to detect a significant difference in the primary outcome.

### 2.7 Statistical analysis

Normally distributed continuous variables were expressed as the mean ± standard deviation or otherwise as the median with range. Categorical variables were expressed as frequencies and percentages. The Shapiro-Wilk test was used to assess normality. Student's *t*-test was used to compare continuous variables with a normal distribution, and for non-normally distributed data Mann-Whitney U was used. The chi-square test was used to analyze categorical data. Odds ratios (ORs) with 95% confidence intervals (CIs) were calculated with binary logistic regression analysis to assess predictors of favorable mRS and hemorrhagic transformation. Propensity score matching (PSM) was conducted using a one-to-one nearest neighbor method without replacement with a caliper width of 0.2, with Cerebrolysin and control as the exposures. The matching covariates included sex, age, risk factors, causes of cardioembolic stroke, baseline NIHSS, mRS, ASPECTS, onset-to-recanalization time, and IV thrombolysis. Following matching, the balance of the sample was evaluated using standardized mean differences (SMD) for included covariates, with acceptable balance defined as a SMD of ≤ 0.1 (32). Additionally, an *E-*value analysis was conducted to evaluate the extent of unmeasured confounding that could potentially nullify our estimates (33). A *p*-value < 0.05 was determined as statistically significant. The analysis was performed using SPSS 27.0 and R software version 4.4.2 (MatchIt and rankinPlot packages, https://www.r-project.org/).

## 3 Results

**Figure 1:**
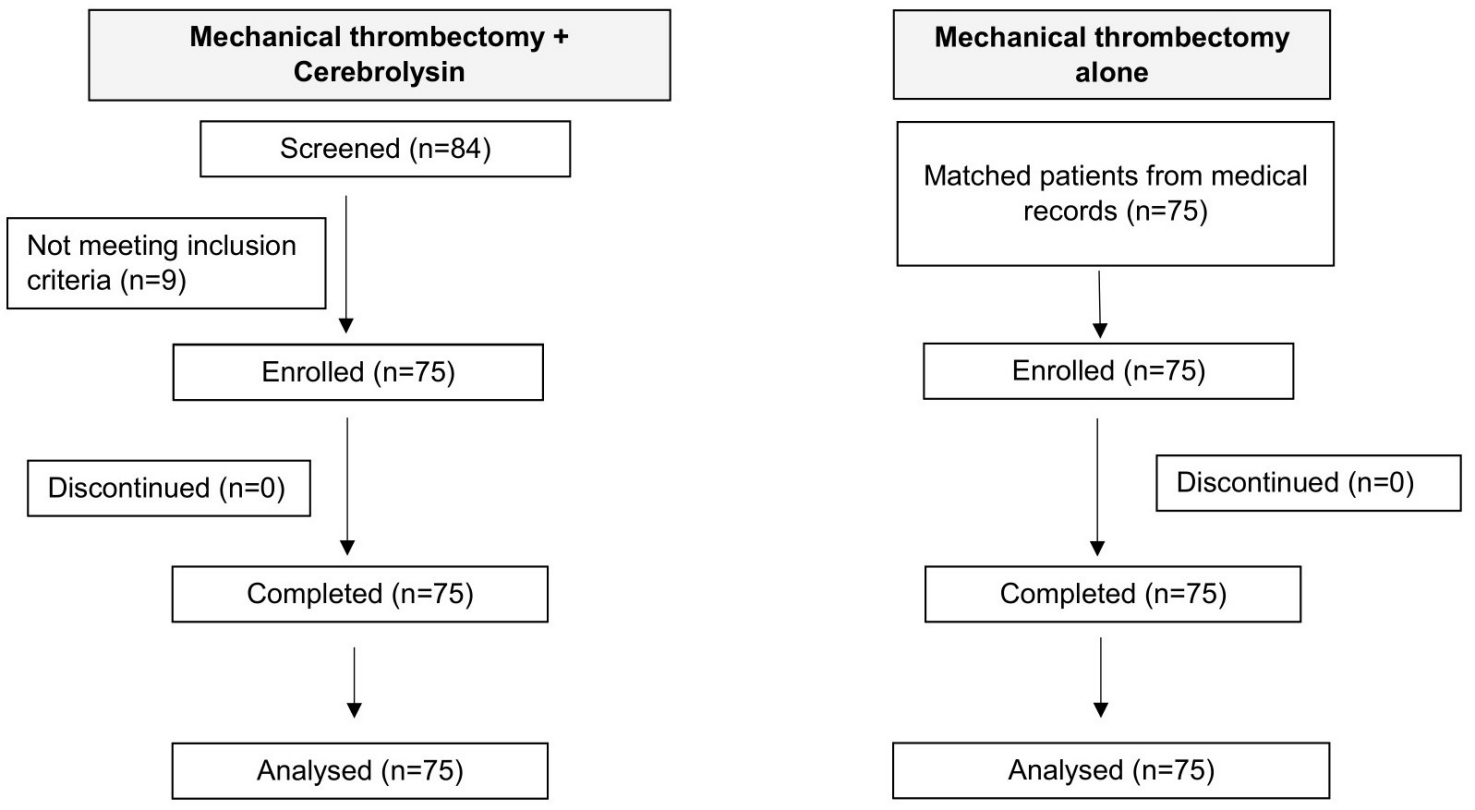
Flowchart of the study.

Of 84 patients who received Cerebrolysin, five cases were excluded due to incomplete data on key variables and four cases due to non-cardioembolic stroke etiology. The final analysis included 75 patients treated with Cerebrolysin after MT, which were well matched with 75 historic controls from the hospital's medical records. The matching criteria included similar occlusion location, age (within ±5 years), co-morbidities, baseline mRS (0–2 or 3–5), initial NIHSS ≥10, ASPECT ≥6, onset to reperfusion time (within ±2 h), and rt-PA administration. Overall, data of 150 patients were analyzed in this study. A flowchart illustrating the patient enrolment is presented in Figure 1.

At baseline, there was a significantly higher proportion of males in the control group (*n* = 45, 60%) compared to the Cerebrolysin group (*n* = 31, 41.3%). The mean age was comparable between both groups, with 59.3 ± 15.5 years in the Cerebrolysin group and 61.8 ± 11.4 years in the control group. Both groups were comparable regarding risk factors, causes of cardioembolic stroke, initial NIHSS, mRS, MoCA, location of occlusion, ASPECT scores, IV thrombolysis, onset to recanalization time, number of retrieval attempts, and TICI ≥2b rates. Peripheral artery diseases were found to be significantly higher in the Cerebrolysin group (12% vs. 0%, *p* = 0.002). After PSM, 51 patients in the Cerebrolysin group were matched with 51 patients in the control group and all covariates demonstrated no statistically significant differences between the two groups. The distribution of propensity scores is shown in Supplementary Figures S1, S2. A summary of baseline characteristics before and after PSM is presented in Table 1.

**Table 1:** Baseline characteristics and risk factors before and after PSM.

**Variables**	**All patients**			**Propensity score-matched patients**			**SMD**
	**MT** + **Cerebrolysin (*****n** =* **75)**	**MT alone (*****n** =* **75)**	***p*** **-value**	**MT** + **Cerebrolysin (*****n** =* **51)**	**MT alone (*****n** =* **51)**	***p*** **-value**	
**Sex, no. (%)**							
Male	31 (41.3)	45 (60)	**0.02** ^*^	23 (45)	25 (49.0)	0.84	−0.08
Female	44 (58.7)	30 (40)	**0.02** ^*^	28 (55)	26 (51)	0.08	0.08
Age, mean ± SD	59.27 ± 15.48	61.76 ± 11.40	0.26	59.45 ± 15.99	60.76 ± 11.95	0.64	−0.04
**Risk factors, no. (%)**							
Smoking	26 (34.7)	25 (33.3)	0.86	18 (35.3)	21 (41.2)	0.54	−0.06
Diabetes mellitus	27 (36)	36 (48)	0.14	20 (39.2)	21 (41.2)	0.84	−0.04
Hypertension	45 (60)	38 (50.7)	0.25	30 (58.8)	28 (54.9)	0.69	0.08
Hyperlipidemia	24 (32)	14 (18.7)	0.06	10 (19.6)	11 (21.6)	0.81	−0.04
Ischemic heart disease	22 (29.3)	21 (28)	0.86	14 (27.5)	14 (27.5)	1	< 0.001
Previous ischemic stroke	9 (12)	9 (12)	1	6 (11.8)	5 (9.8)	0.81	0.06
Peripheral artery disease	9 (12)	0 (0)	**0.002** ^*^	0 (0.0)	0 (0.0)	NA	< 0.001
**Causes of cardioembolic stroke**							
Atrial fibrillation	30 (40)	29 (38.7)	0.87	22 (43.1)	21 (41.2)	0.84	0.04
Mechanical prosthetic valve	10 (13.3)	10 (13.3)	1	5 (9.8)	6 (11.8)	0.75	−0.06
Left ventricle thrombus	7 (9.3)	5 (6.7)	0.76	5 (9.8)	3 (5.9)	0.46	0.09
Dilalted cardiomyopathy	19 (25.3)	18 (24.0)	1	12 (23.5)	12 (23.5)	1	< 0.001
Recent myocardial infarction	3 (4.0)	5 (6.7)	0.72	3 (5.9)	4 (7.8)	0.70	−0.02
Rheumatic heart disease	6 (8)	10 (13.3)	0.07	4 (7.8)	5 (9.8)	0.73	−0.07
Initial NIHSS, median (range)	16 (10–25)	16 (11–25)	0.37	16 (10–25)	16 (11–25)	0.41	−0.25
Initial mRS, median (range)	5 (4–5)	5 (4–5)	0.85	5 (4–5)	5 (4–5)	0.64	−0.09
Initial MoCA, median (range)	23 (21–30)	23 (21–30)	0.11	23 (21–29)	23 (21–29)	0.09	0.02
**Location of occlusion, no. (%)**							
ICA	22 (29.3)	22 (29.3)	0.99	17 (33.3)	13 (25.5)	0.38	0.53
MCA (M1 segment)	46 (65.7)	53 (70.7)	0.23	29 (56.9)	38 (74.5)	0.06	
Basilar and PCA	2 (2.7)	0 (0)	0.16	1 (2)	0 (0.)	0.31	
**ASPECTS**							
6–7	14 (18.7)	14 (18.7)	1	8 (16)	9 (18)	0.79	−0.05
8–10	61 (81.3)	61 (81.3)	1	43 (84)	42 (8)	0.79	0.05
IV thrombolysis	30 (40)	21 (28)	0.12	17 (33.3)	16 (31.4)	0.82	0.02
Onset to recanalization in minutes, median (range)	410 (150–780)	420 (210–910)	0.74	420 (170–780)	420 (210–800)	0.86	0.08
Number of retrieval attempts ≤ 3	58 (77.3)	54 (72)	0.45	38 (74.5)	37 (72.5)	0.82	0.04
TICI ≥2b	66 (88)	61 (81.3)	0.26	44 (86.3)	42 (82.4)	0.91	0.06

SMD, standardized mean difference; ASPECTS, Alberta Stroke Program Early CT Score; mRS, modified Rankin Scale; MT, Mechanical thrombectomy; TICI, Treatment in Cerebral Ischemia; ICA, internal carotid artery; MCA, middle cerebral artery; PCA, posterior cerebral artery; SD, standard deviation; NA, not applicable, Chi square test was used when comparing dichotomous data, Mean (±SD) and Student's t-test was used for normally distributed variables, Median (range) and Mann Whitney test was used for not normally distributed variables, bold values and ^*^p-value < 0.05 was considered statistically significant.

### 3.1 Primary outcome

**Figure 2:**
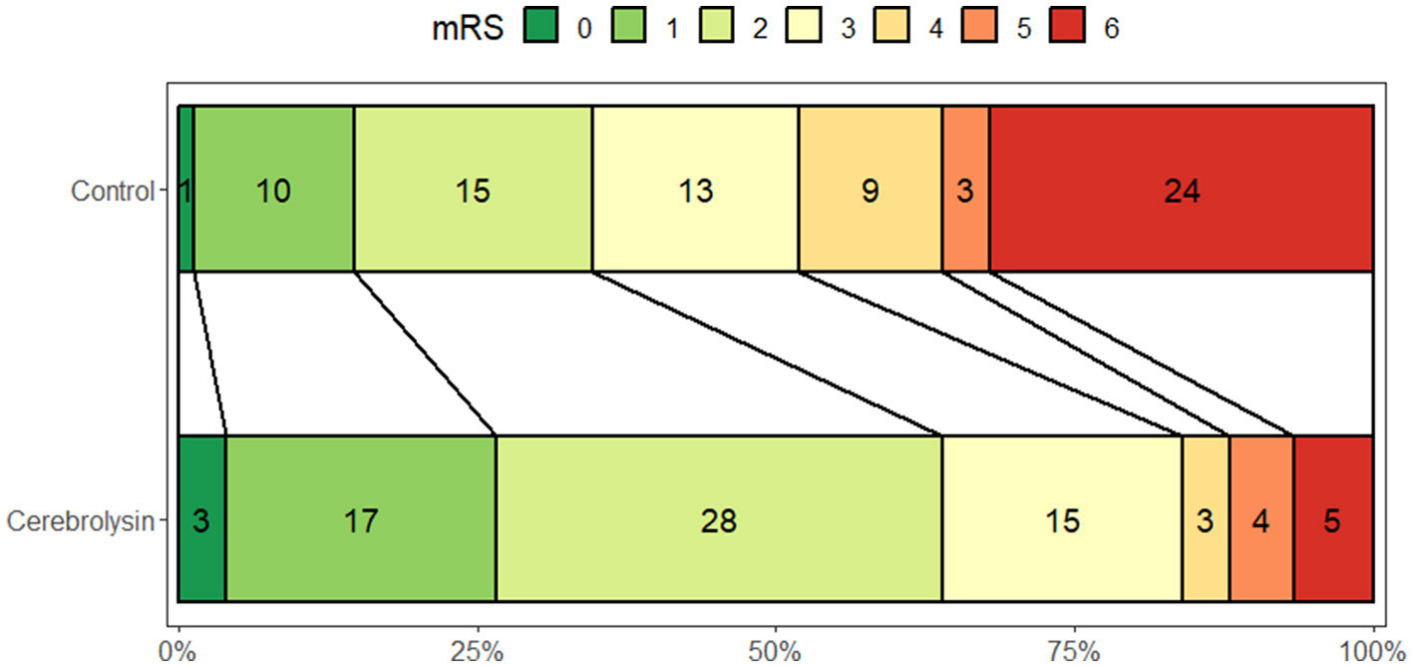
Distribution of the mRS scores at day 90 (*n* = 75 per group).

At day 90, a significantly higher percentage of patients in the Cerebrolysin group achieved a favorable outcome (mRS = 0–2) as compared to the control group (64% vs. 34.7%, *p* < 0.001; Figure 2).

**Figure 3:**
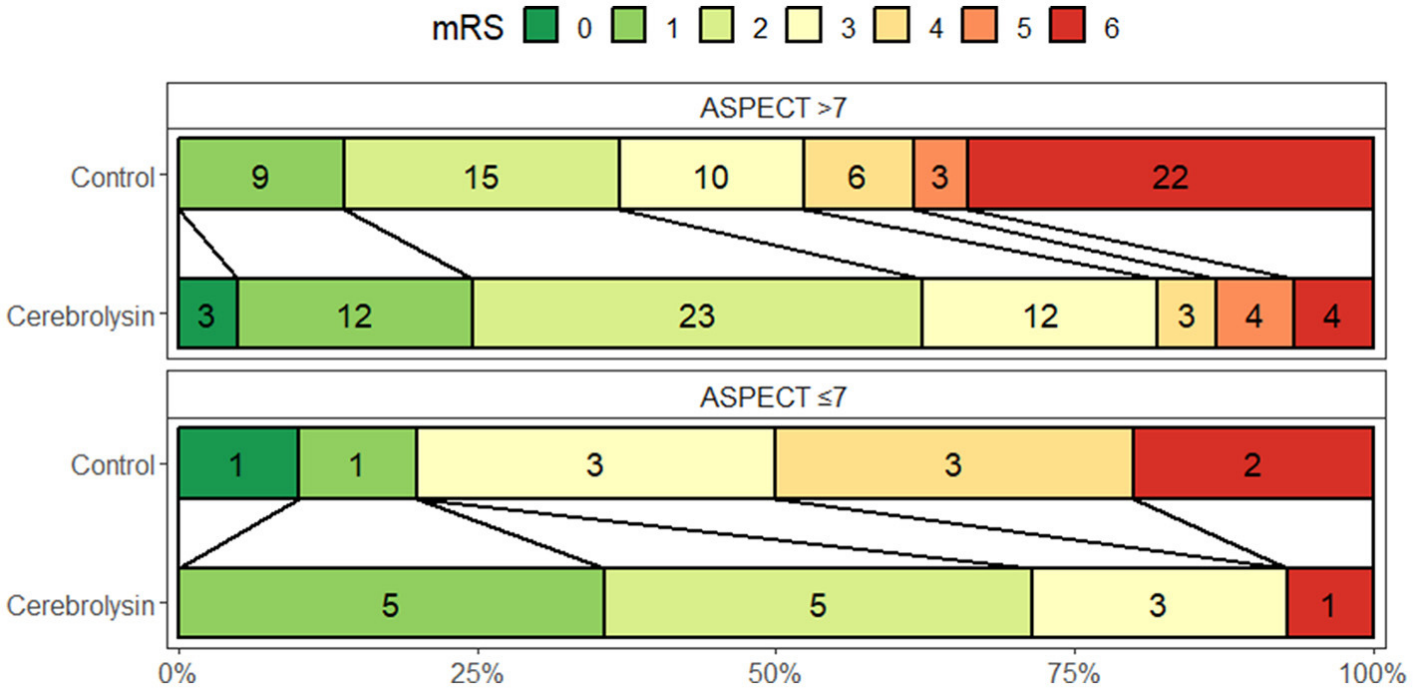
Distribution of the mRS scores at day 90 (*n* = 75 per group) stratified by ASPECTS 8–10 and 6–7.

Subgroup analyses showed that in the Cerebrolysin group 50.7% of patients with ASPECTS of 8–10 achieved a favorable outcome compared to 32% in the control group. In the subgroup of patients with ASPECTS of 6–7, 13.3% of patients in the Cerebrolysin group achieved a favorable outcome as compared to 2.7% in the control group (Figure 3). The results after PSM are consistent with the results of all patients included before PSM, Table 2.

**Table 2:** Functional outcomes at different follow up points before and after PSM.

**Functional outcomes**	**All patients**			**Propensity score-matched patients**		
	**MT** + **Cerebrolysin (*****n** =* **75)**	**MT alone (*****n** =* **75)**	***p*** **-value**	**MT** + **Cerebrolysin (*****n** =* **51)**	**MT alone (*****n** =* **51)**	***p*** **-value**
Favorable mRS at 90 days, *n* (%)	48 (64)	26 (34.7)	**< 0.001** ^*^	33 (65)	16 (31)	**< 0.001** ^*^
mRS at 14 days, median (range)	3 (1–6)	4 (1–6)	**0.02** ^*^	3 (1–6)	4 (1–6)	**0.044** ^*^
mRS at 30 days, median (range)	2 (0–5)	3 (1–6)	**< 0.001** ^*^	3 (0–5)	3 (1–6)	**0.004** ^*^
mRS at 90 days, median (range)	2 (0–6)	3 (0–6)	**< 0.001** ^*^	2 (0–6)	4 (0–6)	**< 0.001** ^*^
NIHSS at 14 days, median (range)	7 (2–19)	10 (2–28)	**< 0.001** ^*^	7 (2–18)	10 (2–28)	**< 0.001** ^*^
NIHSS at 30 days, median (range)	5 (0–17)	8 (1–20)	**0.01** ^*^	5.5 (1–16)	8 (1–20)	**0.02** ^*^
NIHSS at 90 days, median (range)	5 (0–16)	6 (1–16)	**0.02** ^*^	5 (1–15)	6 (1–16)	0.08
MoCA at 90 days, median (range)	30 (23–30)	29.5 (17–30)	0.13	30 (23–30)	30 (17–30)	0.20

NIHSS, National Institutes of Health Stroke Scale; mRS, modified Rankin Scale; MoCA, Montreal Cognitive Assessment; MT, mechanical thrombectomy; Median (range) and Mann Whitney test was used for not normally distributed data, bold values and ^*^p-value < 0.05 was considered statistically significant.

### 3.2 Secondary outcomes

The median mRS scores demonstrated a significant decrease in the Cerebrolysin group at the 2-week, 1 and 3-month time points. Similarly, the median NIHSS scores showed a significant decrease in the Cerebrolysin group at the 2-week, 1 and 3-month time points. However, the MoCA scores at month 3 did not show significant group differences. The results obtained after PSM are consistent with the results of all patients before PSM, except for NIHSS score at 3 months, where no significant difference was observed, Table 2.

**Figure 4:**
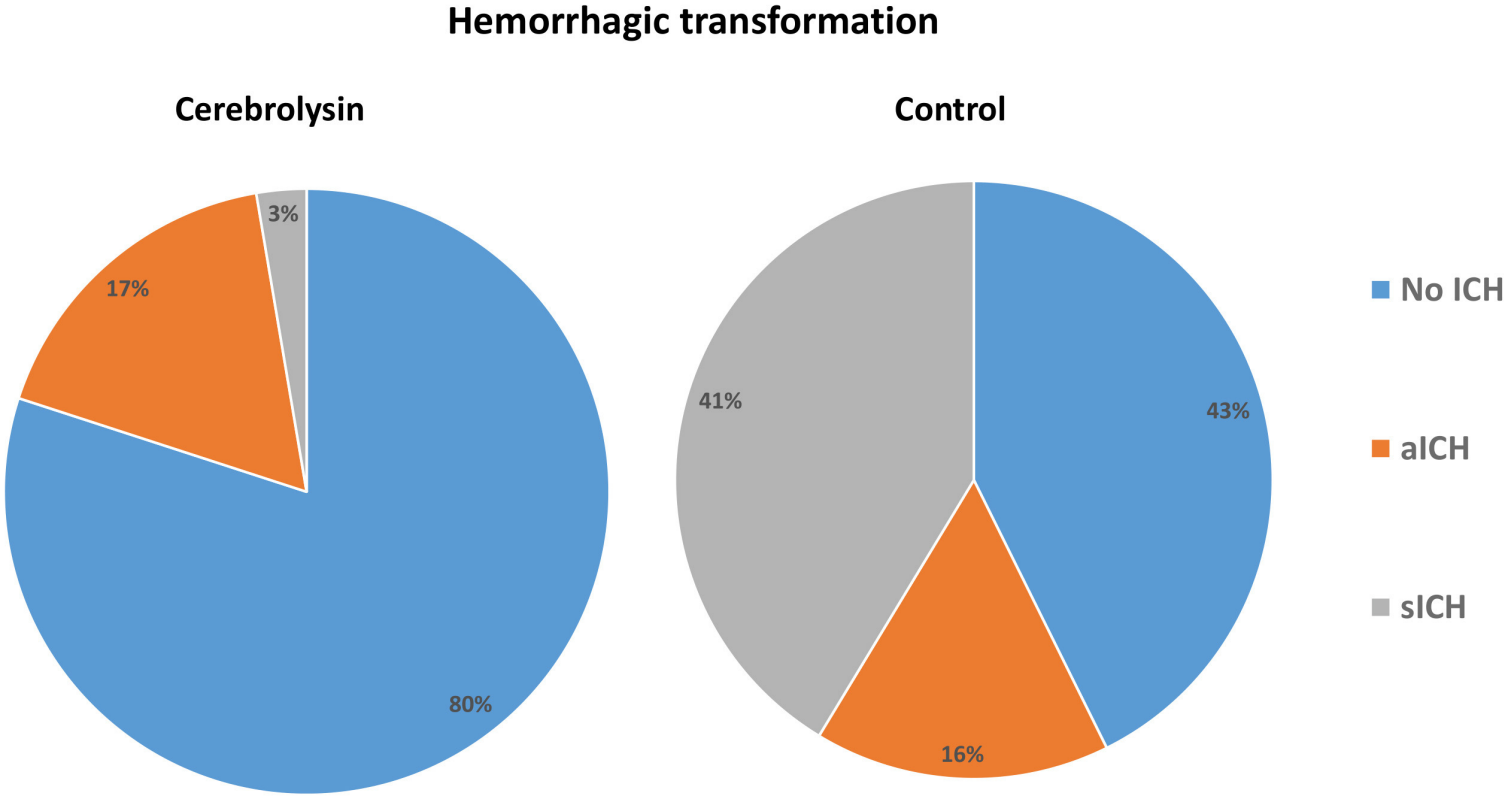
Hemorrhagic transformation rates in the compared groups.

**Figure 5:**
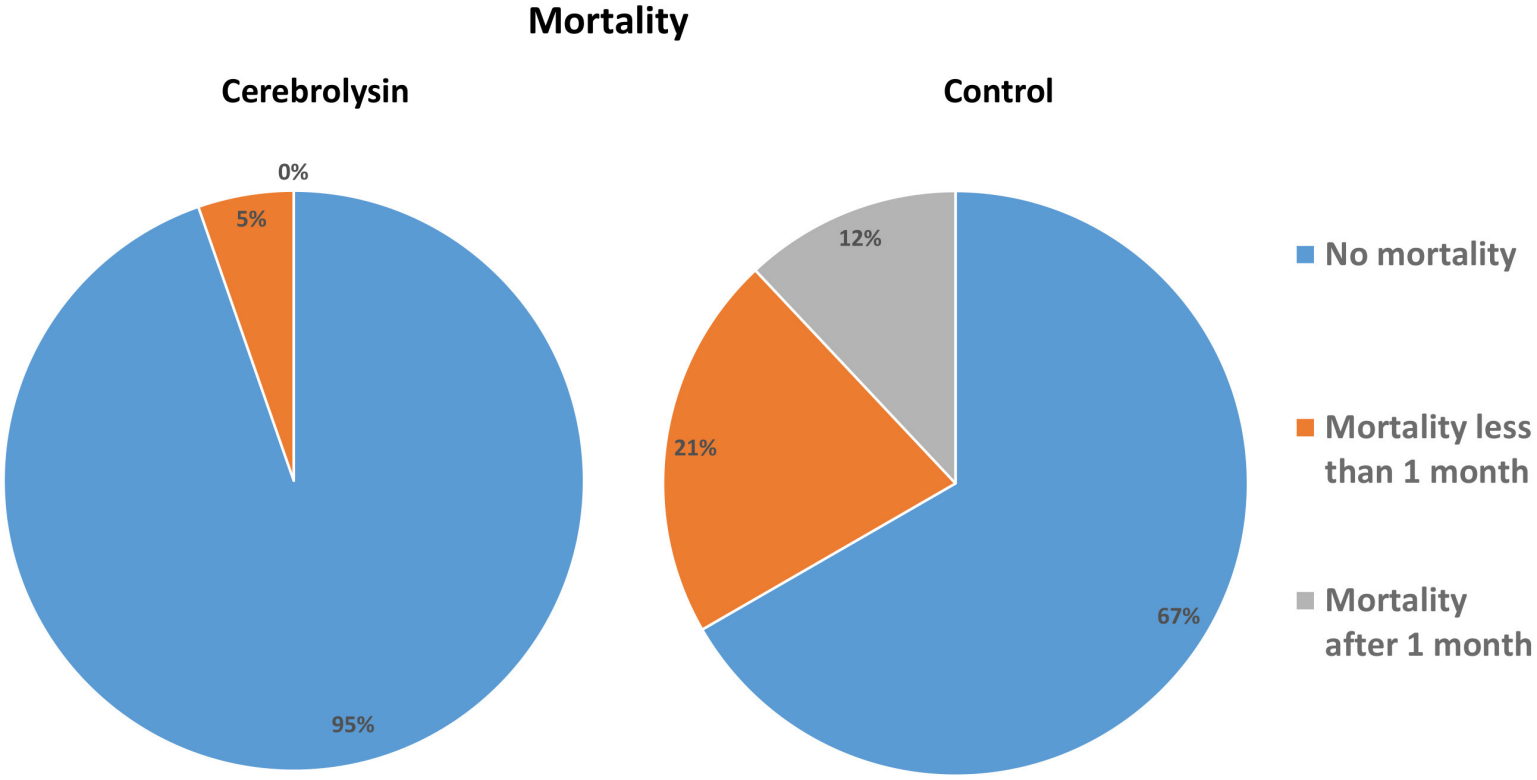
Mortality rates in the compared groups.

The incidences of any hemorrhagic transformation (57.3% vs. 20%) and symptomatic hemorrhage (41.3% vs. 2.7%) were higher in the control group than in the Cerebrolysin group (Figure 4). Furthermore, patients in the control group suffered from higher incidences of PH1 after 24 h, HI2 at day 7, and HI1 and HI2 at day 14. Moreover, the mortality was found to be higher in the control group (32% vs. 5.3%) as was the mortality within the first month (20% vs. 5.3%) and beyond 1 month (12% vs. 0%) (Figure 5). These results were consistent with the findings following PSM analysis, Table 3.

**Table 3:** Hemorrhagic transformation and mortality rates before and after PSM.

**Variables, no. (%)**	**All patients**			**Propensity score-matched patients**		
	**MT** + **Cerebrolysin (*****n** =* **75)**	**MT alone (*****n** =* **75)**	***p*** **-value**	**MT** + **Cerebrolysin (*****n** =* **51)**	**MT alone (*****n** =* **51)**	***p*** **-value**
Any hemorrhagic transformation	15 (20)	43 (57.3)	**< 0.001** ^*^	13 (25.5)	29 (56.9)	**0.001** ^*^
Symptomatic hemorrhagic transformation	2 (2.7)	31 (41.3)	**< 0.001** ^*^	2 (3.9)	22 (43.1)	**< 0.001** ^*^
**Hemorrhagic transformation after 24 h**						
HI1	4 (5.3)	10 (13.3)	0.09	2 (3.9)	6 (11.8)	0.14
HI2	9 (12)	8 (10.7)	0.80	9 (17.6)	5 (9.8)	0.25
PH1	1 (1.3)	21 (28)	**< 0.001** ^*^	1 (2)	16 (31.4)	**< 0.001** ^*^
PH2	1 (1.3)	4 (5.3)	0.17	1 (2)	2 (3.9)	0.56
**Hemorrhagic transformation at day 7**						
HI1	10 (13.3)	14 (18.7)	0.38	10 (19.6)	8 (15.7)	0.60
HI2	1 (1.3)	22 (29.3)	**< 0.001** ^*^	1 (2)	14 (27.5)	**< 0.001** ^*^
PH1	1 (1.3)	3 (4)	0.31	1 (2)	3 (5.9)	0.31
**Hemorrhagic transformation at day 14**						
HI1	0 (0)	6 (8)	**0.048** ^*^	0 (0)	3 (5.9)	0.09
HI2	1 (1.3)	9 (12)	**0.008** ^*^	1 (2)	6 (11.8)	**0.050** ^*^
PH1	0 (0)	2 (2.7)	0.54	0 (0)	2 (3.9)	0.15
Overall mortality	4 (5.3)	24 (32)	**< 0.001** ^*^	3 (5.9)	12 (24)	**< 0.001** ^*^
Mortality < 1 month	4 (5.3)	16 (21.3)	**0.004** ^*^	3 (5.9)	5 (9.8)	0.46
Mortality after 1 month	0 (9)	9 (12)	**0.008** ^*^	0 (0)	7 (13.7)	**0.006** ^*^
**Causes of mortality**						
Hemorrhagic transformation	1 (1.3)	7 (9.3)	**0.03** ^*^	1	7 (13.7)	**0.03** ^*^
Chest infection	2 (2.7)	5 (6.7)	0.25	2 (3.9)	4 (7.8)	0.4
Sepsis	0 (0)	6 (8)	**0.01** ^*^	0 (0)	3 (5.9)	0.09
Sudden death	0 (0)	1 (1.3)	0.32	0 (0)	1 (2)	0.31
Myocardial infarction	1 (1.3)	0 (0)	0.32	0 (0)	0 (0)	NA
Unknown causes	0 (0)	3 (4)	0.08	0 (0)	2 (3.9)	0.15

HI, hemorrhagic infarct; PH, Parenchymal hemorrhage; MT, Mechanical thrombectomy; NA, not applicable. Chi square test was used; Bold values and ^*^p-value < 0.05 was considered statistically significant.

The most prevalent causes of mortality were identified as hemorrhagic transformation and sepsis, with a significantly higher prevalence observed in the control group (Table 3).

### 3.3 Adverse events

The percentages of sepsis (4% vs. 13.3%, *p* = 0.045) and acute kidney injury (0% vs. 5.3%, *p* = 0.043) were significantly less frequent in the MT plus Cerebrolysin group. Following the implementation of the PSM analysis, both groups were found to be comparable with respect to sepsis and acute kidney injury. The occurrence of pneumonia, cardiac ischemia, stroke, infection, hyponatremia, hypernatremia, anemia, deep venous thrombosis, or hypokalemia was comparable in the two groups in the pre- and post-PSM analysis (Table 4).

**Table 4:** Adverse events before and after PSM.

**Adverse events, no. of patients (%)**	**All patients**			**Propensity score-matched patients**		
	**Cerebrolysin (*****n** =* **75)**	**Control (*****n** =* **75)**	***p*** **-value**	**Cerebrolysin (*****n** =* **51)**	**Control (*****n** =* **51)**	***p*** **-value**
Pneumonia	13 (17.3)	16 (21.3)	0.51	11 (21.6)	9 (17.6)	0.62
Sepsis	3 (4.0)	10 (13.3)	**0.045** ^*^	2 (3.9)	6 (11.8)	0.14
Cardiac ischemia	1 (1.3)	1 (1.3)	1	0 (0)	1 (2.0)	0.31
Stroke	1 (1.3)	3 (4.0)	0.31	1 (2.0)	2 (3.9)	0.56
Infection	2 (2.7)	6 (8.0)	0.15	2 (3.9)	5 (9.8)	0.24
Acute kidney injury	0 (1.3)	4 (5.3)	**0.043** ^*^	0 (0)	3 (5.9)	0.08
Hyponatremia	0 (0)	1 (1.3)	0.32	0 (0)	1 (2.0)	0.31
Hypernatremia	1 (1.3)	0 (0)	0.32	1 (2.0)	0 (0)	0.31
Anemia	1 (1.3)	0 (0)	0.32	1 (2.0)	0 (0)	0.31
Deep venous thrombosis	0 (0)	2 (2.7)	0.16	0 (0)	1 (2.0)	0.31
Superficial vein thrombosis	0 (0)	0 (0)	NA	0 (0)	0 (0)	NA
Hypokalemia	0 (0)	1 (1.3)	0.32	0 (0)	1 (2.0)	0.31

NA, not applicable. Chi-Square test was used to compare both groups, bold values and ^*^p-value < 0.05 was considered statistically significant.

### 3.4 Subgroup and sensitivity analyses

In the present study, a multifaceted approach was employed to identify predictors of favorable mRS outcomes and the risk of hemorrhagic transformation. To this end, a binary logistic regression analysis was conducted, with the following stratifications: gender (male vs. female), age (≥70 vs. < 70), baseline ASPECTS (6–7 vs. 8–10), and baseline NIHSS (≤ 17 vs. >17). The results of the logistic regression analysis showed that patients aged 70 and above had a lower likelihood of a favorable mRS and patients with an initial NIHSS scores ≤ 17 had a higher likelihood of a favorable mRS. However, the analysis did not identify age or NIHSS as predictors of a favorable mRS following the PSM analysis. Furthermore, factors such as sex, the use of IV thrombolysis, ASPECTS, and stenting were not identified as predictors for a favorable mRS in either the pre- and post-PSM analyses, as shown in Table 5.

**Table 5:** Adjusted binary logistic regression analysis of factors associated with favorable mRS in the Cerebrolysin group before and after PSM.

**Factors**	**All patients**		**Propensity score-matched patients**	
	**Odds ratio (95% CI)**		**Odds ratio (95% CI)**	***p-*** **value**
Sex (Female vs. male)	0.60 (0.36, 1.01)	0.06	0.16 (0.02, 1.27)	0.08
Age (≥ 70 vs. < 70)	0.48 (0.26, 0.89)	**0.02** ^*^	0.37 (0.07, 2.09)	0.26
IV thrombolysis (yes vs. no)	1.08 (0.60, 1.96)	0.79	1.19 (0.16, 8.70)	0.86
ASPECT (6-7 vs. 8-10)	1.41 (0.49, 4.06)	0.52	6.52 (0.37, 115.64)	0.20
Initial NIHSS (≤ 17 vs. >17)	1.49 (1.002, 2.21)	**0.03** ^*^	5.44 (0.79, 37.67)	0.09
Stenting (yes vs. no)	0.95 (0.69, 1.30)	0.74	0.40 (0.07, 2.20)	0.29

ASPECTS, Alberta Stroke Program Early CT Score; mRS, modified Rankin Scale; MT, Mechanical thrombectomy; NIHSS, National Institutes of Health Stroke Scale; CI, confidence interval; Bold values and ^*^p-value < 0.05 was considered statistically significant.

The application of a logistic regression analysis to the cohort of patients receiving Cerebrolysin showed the presence of predictors of hemorrhagic transformation. Patients exhibiting a favorable mRS were found to be less prone to the development of any such transformation. In the context of the present sample, factors such as sex, age, IV thrombolysis, ASPECT scores, initial NIHSS scores, and stenting were not identified as predictors of hemorrhagic transformation. These findings were consistent with the results following the PSM analysis, as shown in Table 6.

**Table 6:** Adjusted binary logistic regression analysis of factors associated with hemorrhagic transformation in the Cerebrolysin group before and after PSM.

**Factors**	**All patients**		**Propensity score-matched patients**	
	**Odds ratio (95% CI)**	***p*** **-value**	**Odds ratio (95% CI)**	***p*** **-value**
Gender (Female vs. male)	1.32 (0.74, 2.35)	0.29	0.23 (0.21, 2.57)	0.23
Age (≥ 70 vs. < 70)	0.56 (0.31, 1.04)	0.09	5.47 (0.62, 47.98)	0.13
IV thrombolysis (yes vs. no)	0.64 (0.37, 1.13)	0.16	0.14 (0.02, 1.35)	0.09
ASPECT (6–7 vs. 8–10)	0.92 (0.29, 2.88)	0.89	6.97 (0.33, 146.25)	0.21
NIHSS (≤ 17 vs. >17)	1.17 (0.75, 1.82)	0.46	0.45 (0.05, 4.48)	0.51
Favorable vs. unfavorable mRS	2.75 (1.17, 6.45)	**< 0.001** ^*^	0.009 (0.0001, 0.17)	**0.002** ^*^
Stenting (yes vs. no)	1.34 (0.82, 2.21)	0.17	0.31 (0.04, 2.46)	0.27

ASPECTS, Alberta Stroke Program Early CT Score; mRS, modified Rankin Scale; MT, Mechanical thrombectomy; NIHSS, National Institutes of Health Stroke Scale; CI, confidence interval; Bold values and ^*^p-value < 0.05 was considered statistically significant.

*E*-values were calculated in order to assess the robustness of the findings to unmeasured confounding. For the association between Cerebrolysin and mRS, an *E-*value of 6.16 suggests that only a confounder with an extremely strong association (OR ≥ 6.16) with both Cerebrolysin use and mRS could fully explain the observed effect (Figure 3, Supplementary material). A similarly observation was made in the analysis of the association between Cerebrolysin and hemorrhagic transformation, which showed an *E-*value of 10.0, suggesting an even greater degree of robustness (Figure 4, Supplementary material). These results suggest that the findings are unlikely to be nullified by unmeasured confounding.

## 4 Discussion

The development of effective treatment strategies for AIS beyond recanalization is of critical importance. Given that ~80% of strokes are attributable to arterial occlusion, recanalization therapy is imperative for restoring blood flow and reducing stroke-related disability. Nevertheless, it is important to note that only a subset of patients benefits from endovascular therapy (EVT), underscoring the necessity for the development of additional cerebroprotective treatments.

The present study showed a significant improvement in functional outcomes in patients treated with MT ± rt-PA plus Cerebrolysin as compared to MT ± rt-PA alone. The results of the study showed that a higher percentage of patients in the Cerebrolysin group achieved a favorable mRS at day 90, with early recovery evident as early as 2 weeks after stroke.

A novel treatment target for cerebroprotective agents is the enhancement of BBB integrity. As shown in Teng et al. (34), Cerebrolysin has anti-inflammatory effects, reducing the release of pro-inflammatory cytokines, and, consequently, stabilizing the BBB. The present study demonstrates that early administration of Cerebrolysin as an adjunct to MT yielded beneficial outcomes for the rate of symptomatic hemorrhagic transformation. However, it is important to note that Cerebrolysin reduced also the rate of asymptomatic hemorrhagic transformation, a complication that is associated with worse neurological outcome (35). Furthermore, this therapeutic strategy significantly reduced the mortality rate.

The present findings are consistent with the results of the study by Poljakovic et al. in AIS patients with unsuccessful recanalization post-rt-PA. Patients treated with Cerebrolysin (30 ml/day for 14–21 days) showed a notable reduction in hemorrhagic transformation rates and achieved a more favorable outcome (mRS 0–3) at month 12 compared to the control group (70% vs. 48%, *p* = 0.1) (14).

The findings of this study are also consistent with those of the CEREHETIS study by Khasanova (15), which showed that the early administration of Cerebrolysin as an add-on to reperfusion therapy significantly reduced the incidence of symptomatic hemorrhagic transformation and early neurological deficits.

The prolonged and sustained positive effects of Cerebrolysin observed in the patient cohort under investigation are likely due to its neurotrophic and neuroplastic properties, which stimulate neurogenesis and oligodendrogenesis, protecting the neurovascular unit even in the absence of recanalization (12, 36).

With regard to the occurrence of hemorrhagic transformation, the results of the logistic regression analysis indicated that patients exhibiting favorable mRS outcomes were less likely to experience any such transformation. This finding supports the hypothesis that initial recovery outcomes that are more favorable are associated with a reduced risk of post-thrombectomy hemorrhage (37).

With regard to safety and tolerability, the adverse events observed in this study were consistent with those reported in previous Cerebrolysin trials. The types and frequency of symptoms were similar. This finding indicates that Cerebrolysin can be safely administered in conjunction with MT. A meta-analysis of 12 RCTs further confirmed the excellent safety profile of Cerebrolysin (38). An ongoing clinical trial evaluates the efficacy of Cerebrolysin as an adjunct therapy to MT for large vessel occlusion in AIS patients, with symptomatic hemorrhagic transformation and functional outcomes serving as the study endpoints (39).

### 4.1 Strengths

To the best of our knowledge, this research is the first to assess the administration of Cerebrolysin following MT, complemented by a comprehensive assessment of both clinical outcomes and brain imaging. There is a paucity of investigations exploring cerebroprotective substances in combination with MT in cardioembolic AIS. Furthermore, both groups received standard medical care and a PSM was conducted to achieve a more precise estimation of treatment effects, reduce a greater portion of bias, and balance the dataset, enabling direct and effective comparison of baseline covariates between treated and untreated patients (40).

### 4.2 Limitations

The proposed study is subject to several limitations. Firstly, the study incorporates data from the control group, obtained from medical records, which may be subject to selection bias. Secondly, the study was conducted at a single center and included a small sample size. These factors may limit the generalizability of the study's results. Thirdly, the study exhibited limited ethnic and racial diversity, with all participants being of Egyptian origin. Consequently, there is a necessity for additional large-scale clinical studies. Furthermore, although PSM was employed to reduce confounding, it is important to note that retrospective matching is inherently limited in its ability to account for all potential confounding variables. Unmatched or unmeasured variables, such as pre-hospital delays, socioeconomic factors, or operator expertise, could introduce residual bias. Furthermore, temporal differences in clinical practices and diagnostic tools between the historical and treatment groups may also have influenced outcomes.

## 5 Conclusion

The safety of adding Cerebrolysin early to reperfusion therapy was confirmed, and it was found to be associated with significantly improved functional outcomes and reduced symptomatic hemorrhagic transformation and mortality rates. Further prospective studies with larger sample sizes are required to confirm these findings and explore whether extending the treatment period with Cerebrolysin could yield further improvements.

## Data Availability

The raw data supporting the conclusions of this article will be made available by the authors, without undue reservation.
